# Effects of vagal nerve stimulation parameters on heart rate variability in epilepsy patients

**DOI:** 10.3389/fneur.2024.1490887

**Published:** 2024-10-22

**Authors:** Ahmet Genç, Firdevs Ezgi Uçan Tokuç, Meltem Korucuk

**Affiliations:** ^1^Department of Cardiology, Antalya Provincial Health Directorate, Antalya Training and Research Hospital, Antalya, Türkiye; ^2^Department of Neurology, Antalya Provincial Health Directorate, Antalya Training and Research Hospital, Antalya, Türkiye

**Keywords:** vagal nerve stimulation, heart rate variability, drug-resistant epilepsy, 24-h holter electrocardiogram, autonomic nervous system

## Abstract

**Introduction:**

Vagal nerve stimulation (VNS) is used as an alternative treatment in drug-resistant epilepsy patients. Effects of VNS on the cardiac autonomic system are controversial. In this study, we aimed to investigate the relationship between VNS parameters and heart rate variability (HRV) in epilepsy patients who underwent VNS treatment.

**Methods:**

Our study included 31 patients who underwent VNS for drug-resistant epilepsy. Patients were divided into groups according to response to VNS and VNS parameters. All patients underwent 24-h Holter ECG.

**Results:**

The mean age of 31 VNS-treated epilepsy patients included in the study was 33.87 ± 7.6 years. When patients were grouped according to VNS response, 25 patients were in the VNS responder group and six patients were in the VNS-nonresponder group. When comparing Holter parameters in the VNS responder and non-responder groups, the median HF was significantly lower in the VNS responder group. VNS duration and signal frequency had a positive effect on LF/HF, while output and off time had a negative effect on LF/HF. When ROC analysis was performed to determine the cut-off values of the parameters for the VNS-responsive state, the AUC value of the HF parameter was 0.780, which was statistically significant. The cut-off value to distinguish response to VNS was 156.9.

**Conclusion:**

In conclusion, the effects of VNS parameters on HRV parameters are quite complex. However, the conclusion is that VNS is a neuromodulation method that affects the autonomic system in a complex way. Different levels of VNS parameters may also contribute to this effect. Furthermore, HRV parameters can be used as biomarkers to predict the patient population that may benefit from VNS.

## 1 Introduction

Vagal nerve stimulation (VNS) is used as an alternative treatment in drug-resistant epilepsy patients who are not eligible for surgery (1). An implantable device was first approved for epilepsy in Europe in 1994 and in the United States in 1997 (2). Since 1997, more than 120.000 patients, including 30.000 children, have been implanted worldwide (3). Despite this increasing widespread use of VNS, the exact mechanisms of action are not clearly understood (4). While an average of 50% of patients with drug-resistant epilepsy who receive VNS have more than 50% reduction in seizures, approximately one in four patients do not benefit from VNS at all (5). There is no prognostic marker to predict which patients will benefit and how much (6). 80% of the vagus nerve consists of afferent fibers (7). Afferent fibers play an important role in the process of neuromodulation and are probably engaged in the interaction of various cortical networks involved in epileptogenic activity. Efferent fibers innervate the somatic and autonomic nervous systems, with fibers going to both sympathetic and parasympathetic preganglia. In addition, certain efferent fibers also reach the cardiovascular system (8, 9). Vagal efferent fibers innervating the heart induce inhibition of the pacemaker activity of the sinoatrial node. This leads to decreased heart rate, decreased atrioventricular conduction, and decreased excitability of His Purkinje system (10). In the previous studies, it was shown that elevated T wave alternans were observed in patients with drug-resistant epilepsy, and this caused severe interictal cardiac electrical instability, and this instability could be suppressed by VNS. Taking into consideration that T wave alternans are strengthened by sympathetic activation, these findings may suggest that VNS may be related to a decrease in sympathetic tonus (11–13).

We, in this study, aimed to investigate the relationship between VNS parameters and heart rate variability (HRV) in epilepsy patients who underwent VNS treatment.

## 2 Materials and methods

This study was approved by the ethics committee of Antalya Training and Research Hospital (08/06/2023–8/15). Our study included 31 patients who were followed up in the epilepsy outpatient clinic of Antalya Training and Research Hospital with a diagnosis of refractory epilepsy, who were not eligible for surgical treatment after long-term video EEG monitoring and underwent VNS (model 103 neurocybernetic prosthesis; Cyberonics, Pulse Generator, Houston, Texos, United States). Informed consent forms were obtained from all patients and/or their relatives.

Patients included in the study underwent a 24-h holter electrocardiogram (ECG). Demographic data, anti-seizure medication (ASM), and VNS parameters used during Holter ECG were noted.

Patients with other diseases that may affect the cardiac and autonomic nervous systems, those taking any medication other than anti-seizure medication, and alcohol were excluded from the study.

Response to VNS was evaluated based on seizure frequency in the period of 1–3 months before implantation and seizure frequency in the post-implantation period when the patient was included in the study. Patients were divided into two groups: VNS-responder including patients whose seizures decreased by 50% or more after implantation, and VNS-nonresponder including patients whose seizures decreased by less than 50% or not at all (3). The VNS parameters (output, pulse width, signal on time, and signal off time) during the 24-h Holter ECG recording of each patient were noted. Patients were divided into two groups as above and below 2 mA, which were thought to have maximum effect according to the output value (14). Patients were also divided into groups according to pulse width levels (130,250,500 μs,) and signal off time (1.8, 3.5 min).

### 2.1 HRV analysis

In order to assess HRV, 24-h Holter ECG recordings were performed using a three-channel digital Holter recorder DMS300-3A (DM Software Inc., Stateline, NV, United States). Holter recordings were analyzed using Cardioscan 12.0 software (DM Software Inc., Stateline, NV, United States) and recordings were manually evaluated to eliminate artifacts (ectopic beats, arrhythmic events, missing data, and noise effects). All Holter ECG results were evaluated by an experienced cardiologist. Time domain, frequency domain, and nonlinear HRV measures were calculated from the 24 h ECG recordings using recommended methods. SDNN (standard deviation of RR interval normal value 141 ± 39 ms), RMSSD (square root of the mean squared differences of consecutive RR intervals normal values 27 ± 12 ms), and pNN50 (number of interval differences of consecutive RR intervals greater than 50 ms divided by the total number of RR intervals) time domain parameters were evaluated during HRV assessment. Among the time parameters, SDNN is a global predictor of HRV, whereas RMSSD and pNN50 reflect parasympathetic control of heart rate (15). In addition, very low frequency (VLF power between 0.0033 and 0.04 Hz), low frequency (LF power between 0.04 and 0.15 Hz), high frequency (HF power between 0.15 and 0.4 Hz), and LF/HF frequency domain parameters were utilized (15).

Very low frequency, one of the frequency parameters, is associated with prognosis and mortality (16). HF is recognized as a measure of parasympathetic activity and respiratory effect; LF is recognized as a measure of mainly sympathetic activity modulated by the action of the parasympathetic system. The LF/HF ratio expresses the balance between sympathetic and parasympathetic nervous system activity (15).

### 2.2 Statistical method

Data were analyzed with IBM SPSS V23 and IBM AMOS V24. The normality assumption was examined by Shapiro–Wilk test and multiple normality assumption. Yates correction, Fisher’s exact test, and Fisher–Freeman–Halton test were analyzed for the comparison of categorical variables by groups. Mann–Whitney *U* test was used to compare non-normally distributed data according to binary groups. An independent two-sample *t* test was used to compare normally distributed data according to binary groups. One-way ANOVA was used to compare normally distributed data, and Kruskal-Wallis test was used to compare non-normally distributed data according to groups of three or more. Spearman’s rho correlation coefficient was used to examine the relationship between non-normally distributed data. Binary logistic regression analysis was used to examine the effect of independent risk factors on utility. ROC analysis was used to determine the cut-off values of the parameters. Path analysis was used to examine the effect of independent variables on SDNN, rMSSD, LF, HF, VLF, and LF/HF parameters, and Maximum Likelihood method was used as the calculation method. Analysis results were presented as mean ± s. deviation and median (minimum − maximum) for quantitative data and frequency (percentage) for categorical data. The significance level was taken as *p* < 0.050.

## 3 Results

The mean age of 31 VNS-treated epilepsy patients included in the study was 33.87 ± 7.6 years (min: 23, max: 56), and 16 were female and 15 were male. The mean duration of VNS of the patients was 4.38 ± 3.06 years. Demographic data and Holter data of the patients are shown in Table 1. When the patients were classified according to the level of utilization of VNS, 25 patients were in the VNS responder group and six patients were in the VNS-nonresponder group. When Holter parameters were compared in the VNS-responder and VNS-nonresponder groups, the median HF in the VNS-responder group was significantly lower (*p* = 0.035) (Table 2). Patients were classified according to VNS parameters (output, signal off time, and pulse width values), and the relationship with Holter parameters was evaluated. No statistically significant difference was observed (Tables 3–5).

**Table 1:** Descriptive statistics.

	Mean ± s. deviation/Frequency (*n*)	Median (min. − max.)/Percent (%)
Age	33.87 ± 7.6	33 (23.00–56.00)
Gender		
Male	16	51.6
Female	15	48.4
Education		
Illiterate	7	22.6
Primary school	17	54.8
High school	4	12.9
University	3	9.7
Disease period (years)	26.77 ± 8.71	27.50 (14.00–47.00)
Age at disease onset (years)	7.09 ± 6.04	6.00 (0.00–24.00)
Number of ASMs used	4.64 ± 1.05	5.00 (2.00–6.00)
Seizure frequency (month)	8.95 ± 8.9	3.50 (0.00–30.00)
Status epilepticus		
None	20	64.5
Yes	11	35.5
VNS duration (years)	4.38 ± 3.06	3.50 (1–9.00)
Output	1.98 ± 0.65	2.25 (0.75–3.00)
Output		
Below 2	12	38.7
2 and above	19	61.2
Signal on time	32.16 ± 9.56	30.00 (21.00–60.00)
Signal off time	3.45 ± 1.41	3.00 (1.80–5.00)
SDNN	119.03 ± 34.47	115.00 (73.00–194.00)
rMSSD	24.52 ± 9.10	23.00 (11.00–54.00)
PNN50	5.45 ± 5.82	4.00 (0.00–26.00)
LF	560.73 ± 321.73	559.30 (122.70–1216.70)
HF	171.11 ± 134.45	142.60 (19.60–655.00)
VLF	1673.41 ± 985.04	1341.70 (476.80–4800.80)
LF/HF	4.18 ± 2.89	3.59 (0.96–16.81)
Response to VNS		
Yes (VNS-responder)	25	80.6
No (VNS-nonresponder)	6	19.4

VNS, Vagal nerve stimulation; ASM, Anti-seizure medication.

**Table 2:** Comparison of cardiac parameters between VNS-responder and VNS-nonresponder groups.

	VNS-responder		VNS-nonresponder		Test statistics	*p*
	Mean ± s. deviation	Median (min. − max.)	Mean ± s. deviation	Median (min. − max.)		
SDNN (ms)	118.68 ± 33.40	116.00 (73.00–194.00)	120.50 ± 42.05	101.50 (82.00–175.00)	−0.114	0.910**
rMSSD (ms)	23.32 ± 9.01	23.00 (11.00–54.00)	29.50 ± 8.41	32.50 (16.00–37.00)	38.5	0.067*
pNN50 (%)	4.76 ± 5.79	3.00 (0.00–26.00)	8.3 ± 5.47	9.00 (1.00–14.00)	42.5	0.105*
LF (ms^2^)	531.74 ± 335.21	512.40 (122.70–1216.70)	681.52 ± 244.94	715.70 (292.20–959.40)	−1.025	0.314**
HF (ms^2^)	151.83 ± 132.23	140.00 (19.60–655.00)	251.43 ± 122.15	248.35 (81.20–422.90)	33	0.035*
VLF (ms^2^)	1566.78 ± 868.76	1241.60 (476.80–3421.50)	2117.72 ± 1380.34	1610.55 (1053.70–4800.80)	54	0.314*
LF/HF	4.46 ± 3.13	3.78 (0.96–16.81)	3.02 ± 1.00	3.35 (1.64–4.18)	54	0.314*

*Mann–Whitney *U* test, **Independent two sample *t* test.

VNS, Vagal nerve stimulation.

**Table 3:** Comparison of cardiac parameters according to pulse with groups.

	Pulse width					
	130		250		500	
	Mean ± s. deviation	Median (min. − max.)	Mean ± s. deviation	Median (min. − max.)	Mean ± s. deviation	Median (min. − max.)
SDNN (ms)	119.00 ± 36.77	119.00 (93.00–145.00)	105.40 ± 22.86	110.00 (82.00–133.00)	121.88 ± 36.76	115.50 (73.00–194.00)
rMSSD (ms)	23.00 ± 0.00	23.00 (23.00–23.00)	22.20 ± 4.76	23.00 (16.00–29.00)	25.13 ± 10.12	24.00 (11.00–54.00)
pNN50 (%)	3.50 ± 0.71	3.50 (3.00–4.00)	3.20 ± 2.86	3.00 (1.00–8.00)	6.08 ± 6.39	4.00 (0.00–26.00)
LF (ms^2^)	341.80 ± 289.63	341.80 (137.00–546.60)	489.60 ± 274.26	600.40 (123.60–797.30)	593.79 ± 334.48	594.45 (122.70–1216.70)
HF (ms^2^)	107.80 ± 49.21	107.80 (73.00–142.60)	138.66 ± 74.69	119.80 (71.80–254.60)	183.14 ± 147.56	150.30 (19.60–655.00)
VLF (ms^2^)	2240.75 ± 1669.83	2240.75 (1060.00–3421.50)	1543.98 ± 761.31	1768.00 (490.10–2355.30)	1653.10 ± 1005.75	1312.40 (476.80–4800.80)
LF/HF	4.22 ± 4.62	4.22 (0.96–7.49)	3.62 ± 1.88	3.59 (1.72–6.66)	4.30 ± 3.04	3.65 (1.64–16.81)

*Since the sample size of the 130 group was not suitable for comparison, no comparison could be made.

**Table 4:** Comparison of cardiac parameters according to signal off time groups.

	Signal off time						Test statistics	*p*
	1.8		3		5			
	Mean ± s. deviation	Median (min. − max.)	Mean ± s. deviation	Median (min. − max.)	Mean ± s. deviation	Median (min. − max.)		
SDNN (ms)	124.90 ± 36.66	118.00 (75.00–194.00)	119.63 ± 38.40	112.50 (73.00–172.00)	114.15 ± 32.35	100.00 (73.00–186.00)	0.263	0.771**
rMSSD (ms)	27.50 ± 12.65	26.50 (11.00–54.00)	23.13 ± 6.94	22.50 (14.00–35.00)	23.08 ± 6.93	23.00 (13.00–35.00)	0.782	0.467**
pNN50 (%)	7.30 ± 7.99	6.00 (0.00–26.00)	4.38 ± 4.66	3.00 (0.00–13.00)	4.69 ± 4.46	4.00 (0.00–14.00)	0.478	0.787*
LF (ms^2^)	680.59 ± 390.76	715.90 (122.70–1216.70)	444.60 ± 313.83	387.30 (123.60–836.20)	539.98 ± 255.69	546.60 (245.60–1086.70)	1.264	0.298**
HF (ms^2^)	209.19 ± 196.61	141.85 (32.50–655.00)	141.11 ± 94.54	141.40 (19.60–278.70)	160.27 ± 95.12	165.90 (42.80–341.90)	0.173	0.917**
VLF (ms^2^)	1961.09 ± 1172.33	1740.25 (476.80–4800.80)	1451.43 ± 954.20	1037.45 (490.10–3058.20)	1588.72 ± 868.50	1283.10 (782.30–3421.50)	2.057	0.358**
LF/HF	4.28 ± 2.10	3.98 (1.86–7.99)	4.51 ± 5.11	2.92 (0.96–16.81)	3.90 ± 1.47	3.62 (1.4–7.49)	1.425	0.490*

*Kruskal-Wallis test, **One-way ANOVA.

**Table 5:** Comparison of cardiac parameters according to output groups.

	Output				Test statistics	*p*
	Below 2		2 and above			
	Mean ± s. deviation	Median (min. − max.)	Mean ± s. deviation	Median (min. − max.)		
SDNN (ms)	112.75 ± 34.99	99.50 (73.00–186.00)	123.00 ± 34.48	116.00 (75.00–194.00)	−0.802	0.429**
rMSSD (ms)	23.42 ± 7.28	23.00 (14.00–35.00)	25.21 ± 10.22	23.00 (11.00–54.00)	−0.528	0.602**
pNN50 (%)	5.00 ± 4.71	4.00 (0.00–14.00)	5.74 ± 6.53	3.00 (0.00–26.00)	113.5	0.984*
LF (ms^2^)	541.60 ± 258.97	529.50 (291.40–1086.70)	572.81 ± 362.12	629.60 (122.70–1216.70)	−0.259	0.798**
HF (ms^2^)	159.93 ± 103.83	141.50 (19.60–341.90)	178.17 ± 152.98	142.60 (32.50–655.00)	111	0.921*
VLF (ms^2^)	1624.53 ± 952.61	1175.00 (662.30–3421.50)	1704.28 ± 1029.57	1497.70 (476.80–4800.80)	109	0.857*
LF/HF	4.82 ± 4.04	3.74 (1.64–16.81)	3.77 ± 1.87	3.11 (0.96–7.99)	102	0.646*

*Mann–Whitney *U* test, **Independent two sample *t* test.

**Figure 1:**
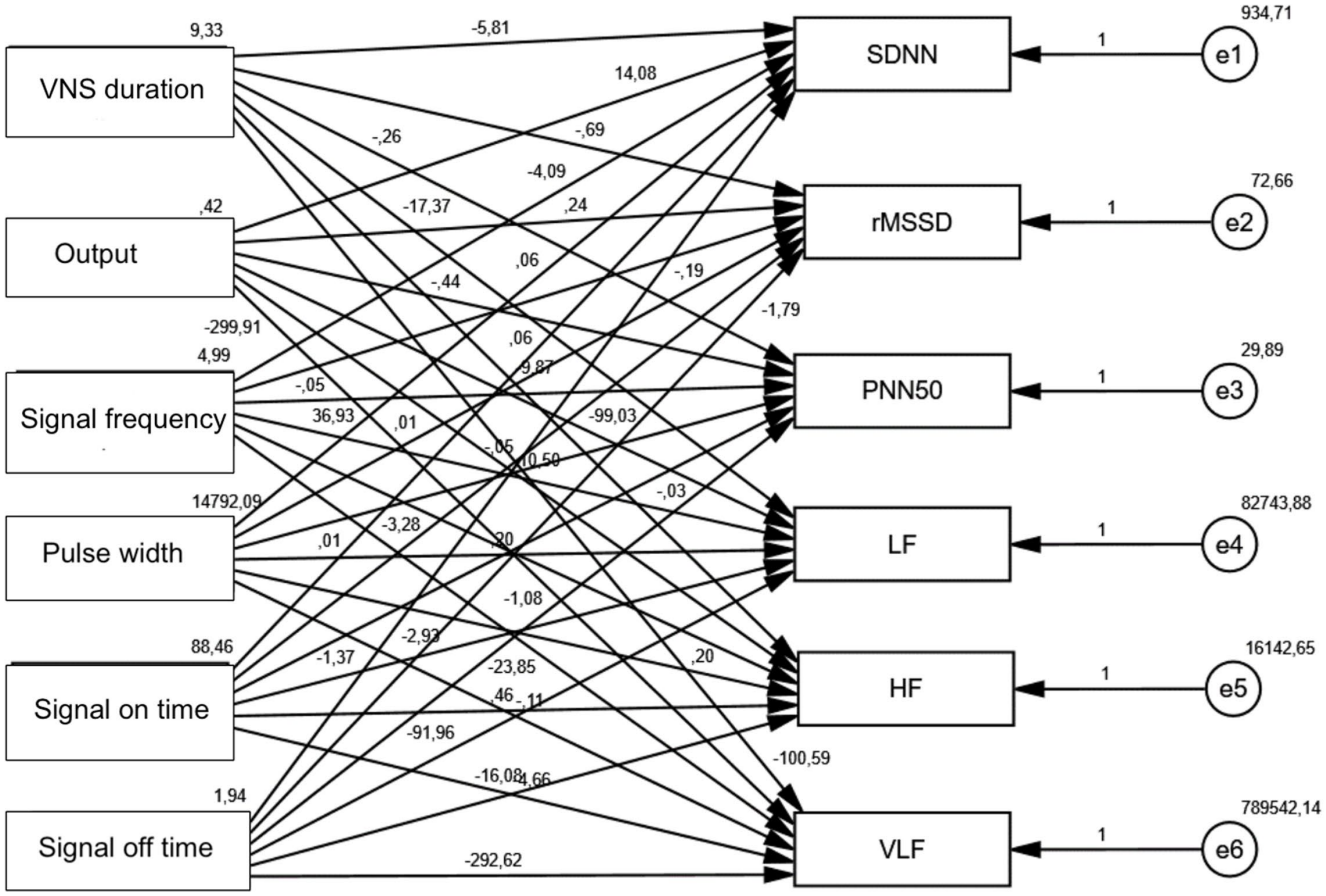
Non-standardized beta coefficients.

**Figure 2:**
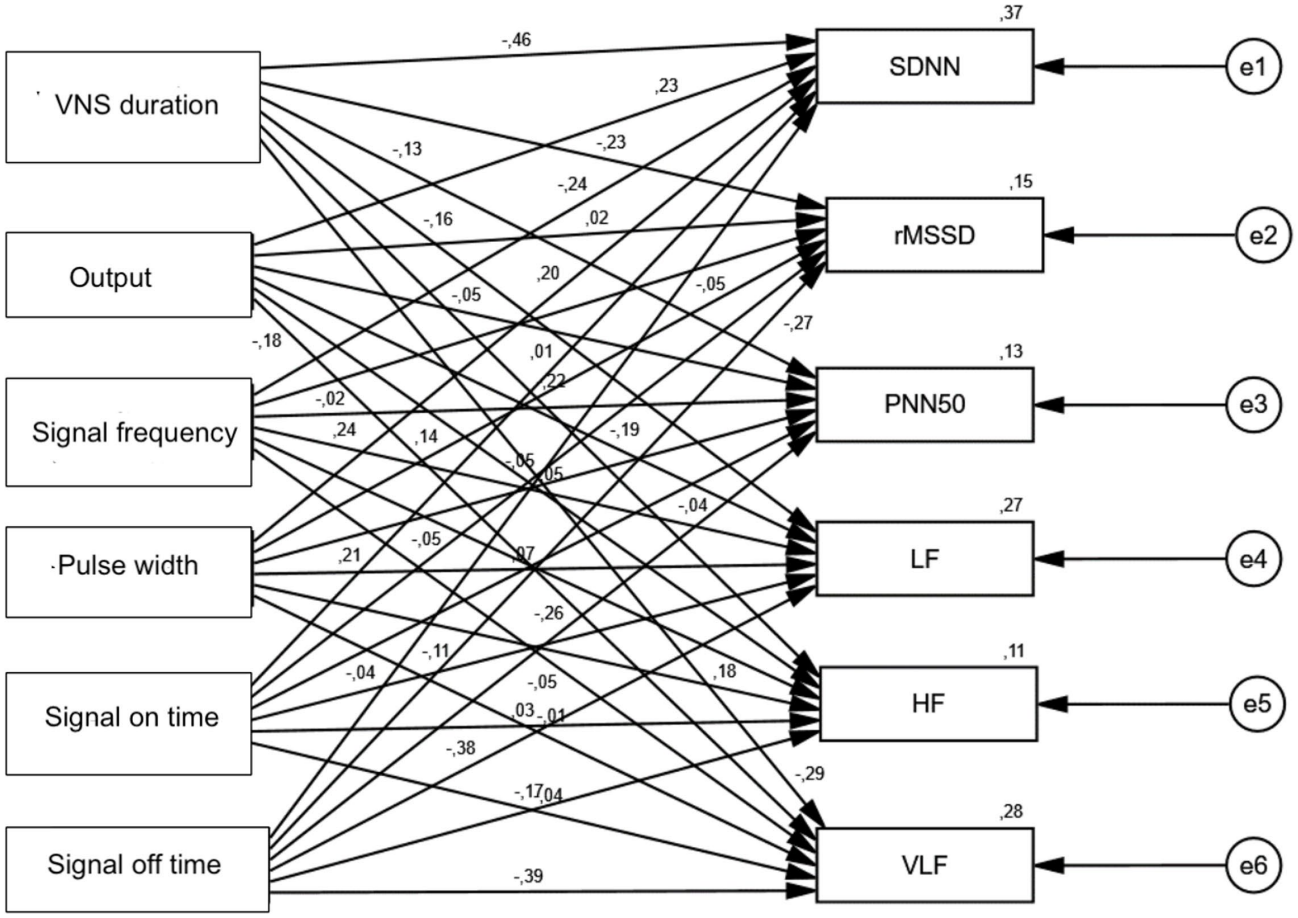
Standardized beta coefficients.

When path analysis of the effects of VNS duration, Output, Signal frequency, Pulse width, signal on time, and signal off time variables on SDNN, rMSSD, PNN50, LF, HF, and VLF values was performed, a statistically significant negative effect of VNS duration variable on SDNN was observed (*p* = 0.001). A statistically significant negative effect of signal off time on LF was observed (*p* = 0.015). A statistically significant negative effect of signal off time on VLF was observed (*p* = 0.012) (Table 6; Figures 1, 2).

**Table 6:** Examining the effects of independent variables on SDNN, rMSSD, LF, HF, and VLF by path analysis.

Dependent variable		Independent variable	β1	β2	S. fault	Test statistics	*p*	*R*^2^
SDNN	<−--	VNS duration	−0.460	−5.813	1.827	−3.182	0.001	0.374
SDNN	<−--	Output	0.235	14.082	8.664	1.625	0.104	
SDNN	<−--	Signal frequency	−0.237	−4.092	2.498	−1.638	0.101	
SDNN	<−--	Pulse width	0.201	0.064	0.046	1.390	0.164	
SDNN	<−--	Signal on time	0.015	0.060	0.593	0.101	0.919	
SDNN	<−--	Signal off time	−0.106	−2.931	4.010	−0.731	0.465	
rMSSD	<−--	VNS duration	−0.229	−0.693	0.509	−1.360	0.174	0.148
rMSSD	<−--	Output	0.017	0.242	2.416	0.100	0.920	
rMSSD	<−--	Signal frequency	−0.047	−0.193	0.696	−0.277	0.782	
rMSSD	<−--	Pulse width	0.135	0.010	0.013	0.802	0.422	
rMSSD	<−--	Signal on time	−0.047	−0.047	0.165	−0.281	0.779	
rMSSD	<−--	Signal off time	−0.270	−1.790	1.118	−1.601	0.109	
PNN50	<−--	VNS duration	−0.134	−0.258	0.327	−0.789	0.430	0.132
PNN50	<−--	Output	−0.048	−0.438	1.549	−0.282	0.778	
PNN50	<−--	Signal frequency	−0.020	−0.053	0.447	−0.120	0.905	
PNN50	<−--	Pulse width	0.210	0.010	0.008	1.234	0.217	
PNN50	<−--	Signal on time	−0.043	−0.027	0.106	−0.253	0.801	
PNN50	<−--	Signal off time	−0.256	−1.079	0.717	−1.505	0.132	
LF	<−--	VNS duration	−0.157	−17.369	17.190	−1.010	0.312	0.271
LF	<−--	Output	−0.189	−99.028	81.517	−1.215	0.224	
LF	<−--	Signal frequency	0.245	36.931	23.499	1.572	0.116	
LF	<−--	Pulse width	0.071	0.197	0.432	0.455	0.649	
LF	<−--	Signal on time	−0.038	−1.366	5.584	−0.245	0.807	
LF	<−--	Signal off time	−0.380	−91.963	37.725	−2.438	0.015	
HF	<−--	VNS duration	−0.223	−9.870	7.593	−1.300	0.194	0.115
HF	<−--	Output	0.050	10.501	36.006	0.292	0.771	
HF	<−--	Signal frequency	−0.054	−3.285	10.379	−0.316	0.752	
HF	<−--	Pulse width	0.176	0.195	0.191	1.025	0.306	
HF	<−--	Signal on time	0.032	0.459	2.466	0.186	0.852	
HF	<−--	Signal off time	−0.166	−16.079	16.663	−0.965	0.335	
VLF	<−--	Output	−0.185	−299.907	251.808	−1.191	0.234	0.277
VLF	<−--	Signal frequency	−0.051	−23.853	72.589	−0.329	0.742	
VLF	<−--	Pulse width	−0.013	−0.109	1.334	−0.082	0.935	
VLF	<−--	Signal on time	−0.042	−4.664	17.249	−0.270	0.787	
VLF	<−--	Signal off time	−0.390	−292.617	116.534	−2.511	0.012	
VLF	<−--	VNS duration	−0.294	−100.592	53.101	−1.894	0.058	

β1, Standardized beta coefficient; β2, Unstandardized beta coefficient.

VNS, Vagal nerve stimulation.

**Figure 3:**
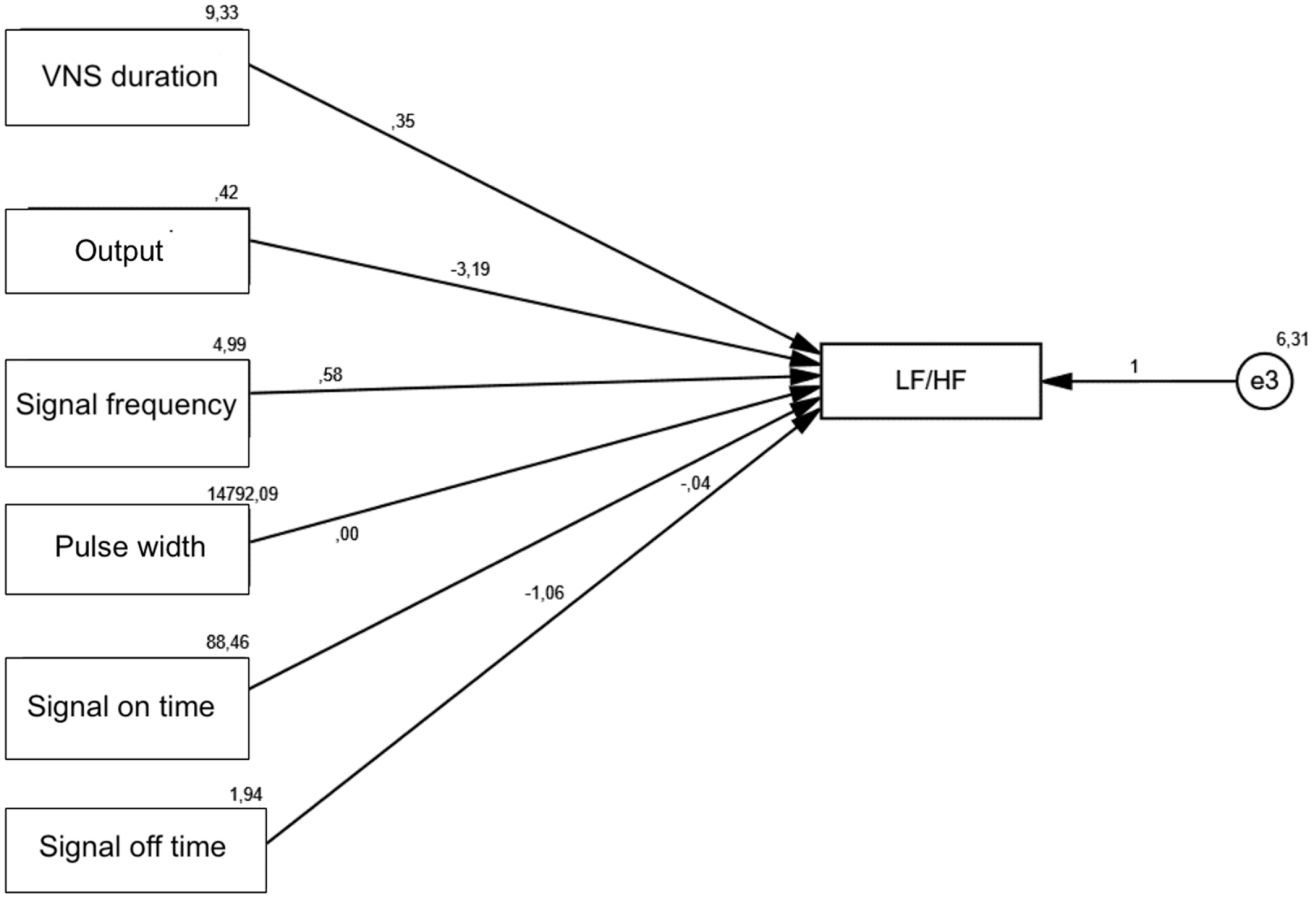
Non-standardized beta coefficients.

**Figure 4:**
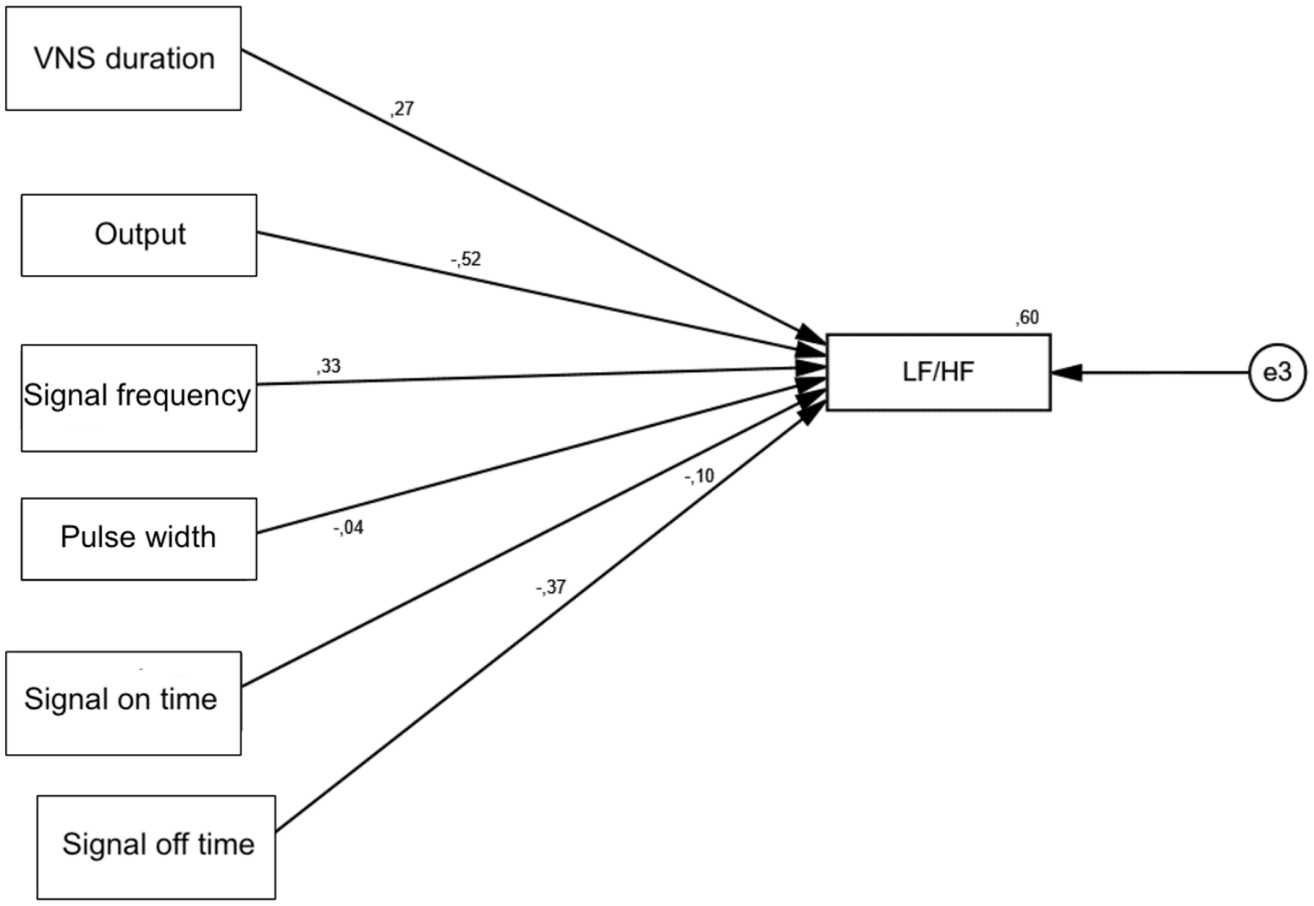
Standardized beta coefficients.

In addition, a statistically significant positive effect of VNS duration variable on LF/HF was found (*p* = 0.021). Signal frequency was detected to have a statistically significant positive effect on LF/HF (*p* = 0.005). The output variable was found to have a statistically significant negative effect on LF/HF (*p* < 0.001). The signal off time variable was found to have a statistically significant negative effect on LF/HF (*p* = 0.001) (Table 7; Figures 3, 4).

**Table 7:** Examining the effects of independent variables on LF/HF by path analysis.

Dependent variable		Independent variable	β1	β2	S. fault	Test statistics	*p*	*R*^2^
LF/HF	<−--	VNS duration	0.268	0.348	0.15	2.316	0.021	0.598
LF/HF	<−--	Output	−0.518	−3.19	0.712	−4.48	<0.001	
LF/HF	<−--	Signal frequency	0.325	0.576	0.205	2.808	0.005	
LF/HF	<−--	Pulse width	−0.044	−0.001	0.004	−0.382	0.703	
LF/HF	<−--	Signal on time	−0.102	−0.043	0.049	−0.879	0.379	
LF/HF	<−--	Signal off time	−0.374	−1.064	0.33	−3.23	0.001	

β1, Standardized beta coefficient; β2, Unstandardized beta coefficient.

VNS, Vagal nerve stimulation.

**Figure 5:**
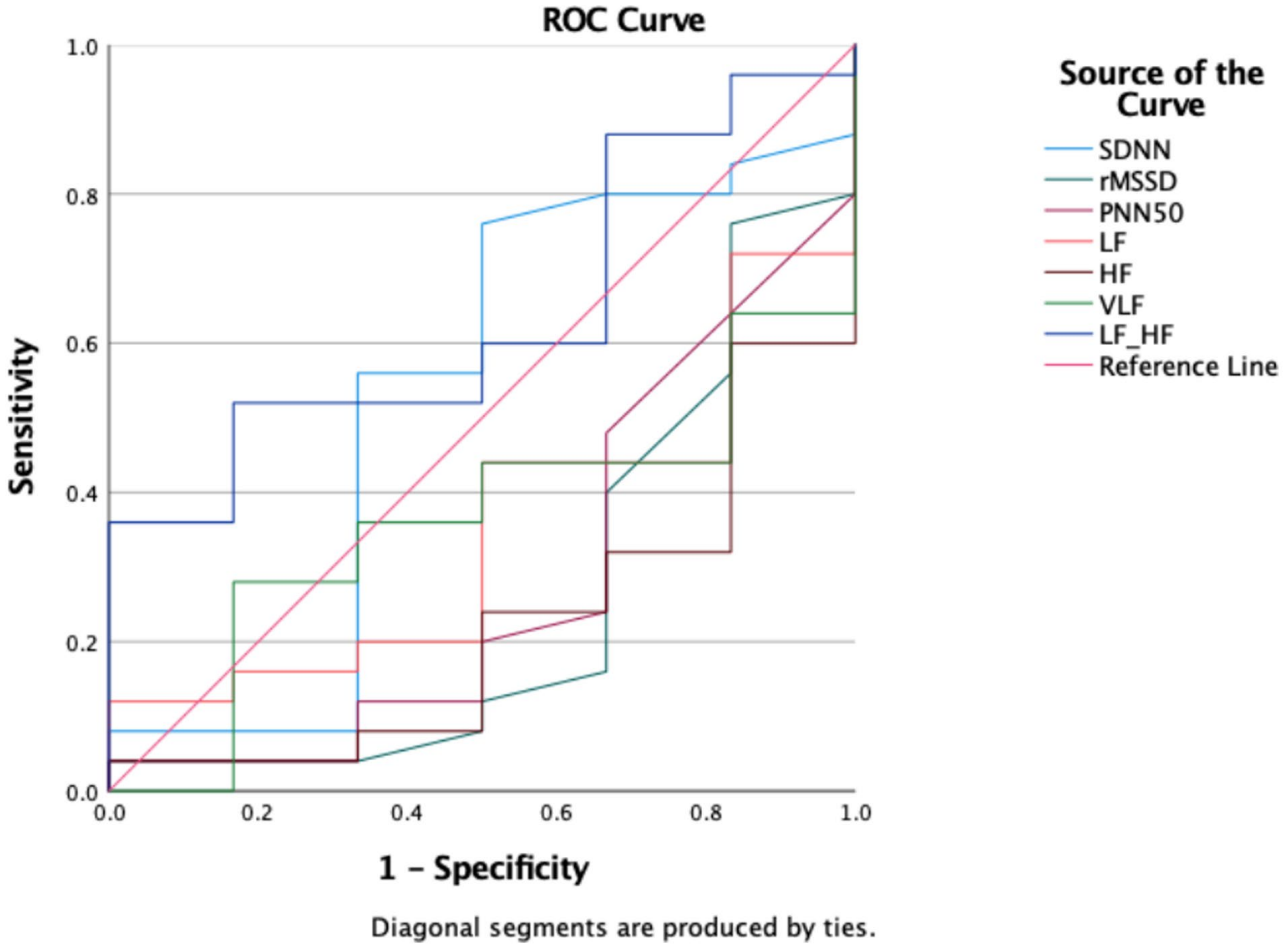
ROC curve of parameters for the benefit status.

ROC analysis was performed to determine the cut-off values of the parameters for VNS-responder status. As a result of the analysis, the AUC value of the HF parameter was 0.780, which was statistically significant (*p* = 0.036). The cut-off value for discriminating response to VNS was 156.9. The sensitivity, specificity, PPV, PPV, and NPV of the cut-off value were 68, 83.3, 94.4, and 38.5%, respectively (Table 8; Figure 5). Since the AUC values of the other parameters were not significant, no cut-off value was calculated (*p* > 0.050).

**Table 8:** Determination of cut-off values for parameters for the benefit status.

	Cut-off value	AUC (%95 CI)	*p*	Sensitivity (%)	Specificity (%)	PPV (%)	NPV (%)
SDNN	---	0.473 (0.186–0.761)	0.841	---	---	---	---
rMSSD	---	0.743 (0.5–0.987)	0.068	---	---	---	---
PNN50	---	0.717 (0.48–0.953)	0.104	---	---	---	---
LF	---	0.653 (0.436–0.871)	0.250	---	---	---	---
HF	≤156.9	0.78 (0.587–0.973)	0.036	68%	83.3%	94.4%	38.5%
VLF	---	0.64 (0.427–0.853)	0.294	---	---	---	---
LF/HF	---	0.36 (0.141–0.579)	0.294	---	---	---	---

PPV, Positive predictive value; NPV, Negative predictive value.

## 4 Discussion

The effects of VNS treatment on cardiovascular autonomic functions in individuals with epilepsy are highly controversial. Although some groups have argued that VNS administration has no effect on cardiac autonomic functions, recent studies have shown its clear effects on HRV (17–22). When the factors affecting HRV in patients undergoing VNS are examined, studies conducted especially between groups responding and not responding to VNS administration come to the forefront (6, 20).

However, only a few studies have previously investigated the relationship between VNS parameters and HRV. Several studies have examined HRV parameters between VNS on and off periods and have shown highly contradictory results. Some studies have found that HF and LF power increased in the VNS on period, while some studies have found that HF power decreased (19, 23–25). In a systematic review including 229 epilepsy patients from 20 studies investigating HRV effects after VNS administration, factors affecting HRV were examined, and it was observed that the HF heart rate variable was lower preoperatively than postoperatively in the VNS-responder subgroup. In addition, when meta-regression analyses of VNS parameters and HRV parameters of all patients were performed, it was noted that VNS parameters did not show any effect on HRV parameters (18). To the best of our knowledge, studies on VNS parameters in epilepsy patients are limited to these studies. This is a very significant issue that has not been paid much attention to before. Because the optimum VNS parameter setting that can minimize seizures by considering side effects may be different for each patient. From this perspective, we studied all VNS parameters that we thought might affect HRV parameters in a multimodel. Our study is one of the rare and comprehensive studies evaluating the effect of VNS parameters on HRV. One of the most remarkable results of our study is the effects of VNS parameters, especially signal off time, signal on time, signal frequency and output parameters on HRV.

The effects of transcutaneous VNS parameters on autonomic functions using HRV were investigated in 48 healthy participants. Participants were first given short-term and then long-term stimulations. An increase in LF and LF/HF ratio was observed after prolonged stimulation. In addition, in this study, pulse width and frequency, which are VNS parameters, were shown to cause a change in the LF/HF ratio and based on these results, it was argued that t-VNS contributes to changing HRV in healthy population (26). In another study, lower LF/HF rates were observed when in VNS on period (27). The results of our study also show that VNS parameters affect LF and LF/HF change and HRV in epilepsy patients. Although the physiologic interpretation of the VLF band is not as clear as HF and LF, it is considered to be associated with prognosis and mortality compared to the others (17, 28).

However, when the literature is reviewed, there is no clear information on the VLF band in epilepsy patients undergoing VNS. Previous studies have shown that VNS reduces the risk of mortality and SUDEP (29). If we associate the VLF band with mortality, the inverse correlation between signal off time and VLF observed in our study may explain why VNS administration reduces the risk of SUDEP and mortality.

Another remarkable finding in our study is that HF may be a non-invasive parameter that can be used for the benefit status of VNS administration as a result of ROC analyses. In a previous study evaluating HRV parameters before and after VNS, the RMSSD value was found to have high sensitivity and specificity in terms of detecting VNS responders (6). Similar results were obtained in a study by Liu et al. (30). This information is important. Since VNS treatment utilized in patients with drug-resistant epilepsy is a highly expensive treatment, it is particularly challenging to access in developing and underdeveloped countries (31–33). Predicting the VNS response using these Parameters will facilitate patient selection.

There are some limitations of our study. The number of samples we have is relatively small. In addition, inclusion of a healthy control patient group in the study and performing repeated Holter applications before VNS and at routine intervals in patients undergoing VNS may have increased the power of the study.

In conclusion, the effects of VNS parameters on HRV parameters are quite complex. However, the conclusion is that VNS is a neuromodulation method that affects the autonomic system in a complex way. Different levels of VNS parameters may also contribute to this effect. Furthermore, HRV parameters can be used as biomarkers to predict the patient population that may benefit from VNS. Large sample, prospective, long-term double-blind studies are needed in this regard.

## Data Availability

The original contributions presented in the study are included in the article/supplementary material, further inquiries can be directed to the corresponding author.
